# Experimental Microbiomes: Models Not to Scale

**DOI:** 10.1128/mSystems.00175-19

**Published:** 2019-07-30

**Authors:** Marc G. Chevrette, Jennifer R. Bratburd, Cameron R. Currie, Reed M. Stubbendieck

**Affiliations:** a Department of Bacteriology, University of Wisconsin—Madison, Madison, Wisconsin, USA; b Laboratory of Genetics, University of Wisconsin—Madison, Madison, Wisconsin, USA; University of Pittsburgh Medical Center

**Keywords:** interactions, microbial communities, microbiome, model systems

## Abstract

Low-cost, high-throughput nucleic acid sequencing ushered the field of microbial ecology into a new era in which the microbial composition of nearly every conceivable environment on the planet is under examination. However, static “screenshots” derived from sequence-only approaches belie the underlying complexity of the microbe-microbe and microbe-host interactions occurring within these systems. Reductionist experimental models are essential to identify the microbes involved in interactions and to characterize the molecular mechanisms that manifest as complex host and environmental phenomena. Herein, we focus on three models (*Bacillus*-*Streptomyces*, Aliivibrio fischeri-Hawaiian bobtail squid, and gnotobiotic mice) at various levels of taxonomic complexity and experimental control used to gain molecular insight into microbe-mediated interactions. We argue that when studying microbial communities, it is crucial to consider the scope of questions that experimental systems are suited to address, especially for researchers beginning new projects. Therefore, we highlight practical applications, limitations, and tradeoffs inherent to each model.

## Abstract (precis)

Low-cost, high-throughput nucleic acid sequencing ushered the field of microbial ecology into a new era in which the microbial composition of nearly every conceivable environment on the planet is under examination. However, static “screenshots” derived from sequence-only approaches belie the underlying complexity of the microbe-microbe and microbe-host interactions occurring within these systems.

## PERSPECTIVE

Microbiomes shape the fundamental biology of environments and can have substantial impacts on macroscopic ecosystems. Within their hosts, microbiomes alter metabolism, behavior, and disease. Experimental insight into the molecular mechanisms underlying microbiome interactions remains elusive. High complexity, variable plasticity, and low manipulability of natural systems remain barriers to recapitulating microbiomes in the laboratory.

Distilling the extreme complexity of biology into discrete, functional units remains a difficult challenge. As early as 1662, René Descartes posited that biology could be explained as collectives of self-operating machinery termed “automata” (1). We have dissected the molecular nature of these “machines” into their constituent parts. For example, forward genetic screens, reverse genetics, and complementation aim to connect genomic loci with organism-level effects and are invaluable in understanding how genes function and phenotypes manifest. As we increasingly appreciate how microbes influence ecology and host fitness, models are essential to limit complexity and maximize experimental control, such that we can begin to understand how interactions within microbial communities influence biology (2). From a microbial perspective, understanding the influences of fitness can resolve common and distinct features of microbial interactions in different systems. While microbial fitness is often conceived of as a static property, the dynamics of microbial interactions are shaped by environmental and temporal plasticity and competition. Thus, the phenotypes that shape microbial fitness are the sum of many variables, including but not limited to the presence and regulation of genes, the interspecies interactions of a microbial community, and chemical gradients (3). Further, emergent properties of microbial communities can confound the simplest studies. For example, different combinations of relatively simple ≤5 member communities in *Drosophila* can mediate changes in host life span and fecundity, with some members influencing these traits only in the presence of certain other community members (4). Considering this complexity, model systems integrating reductionist experimental frameworks are necessary to link the underlying interaction networks of microbiomes to host biology.

**FIG 1:**
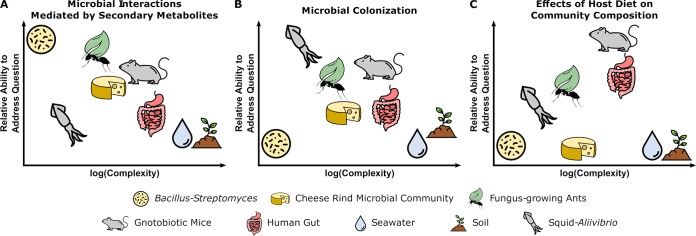
Tradeoffs between experimental questions and complexity of microbiome systems. Each microbiome system is suited to address different types of questions based on the culturability of microbes, genetic tractability of microbes and host (where relevant), ability to maintain system in laboratory setting, and ability to make host/environment germfree. Three different systems are shown in this figure as examples. (A) Pairwise interactions between B. subtilis and *Streptomyces* spp. are well-suited for characterizing the functions of secondary metabolites in microbial interactions. (B) The symbiosis between bobtail squid and *A. fischeri* is fundamental to understanding host and microbial factors that influence colonization. (C) The use of gnotobiotic mice is crucial for making links between host diet and the effects on specific microbial taxa in a community (see the text for specific details). Specific original image credit from the Noun Project (https://thenounproject.com/): Fertile Soil by Ben Davis; Droplet by Focus; Mouse by Iconic; Cheese Wheel by Anniken & Andreas; Bacteria by Arthur Shlain; Squid by Artem Kovyazin; ant by Yugudesign; leaf by Saeful Muslim; all used and modified under the Creative Commons License, Attribution 3.0.

For early career researchers and researchers embarking on new projects, it is important to understand the kinds of questions that certain models address well and where reduction can maximize experimental control with minimal loss of biological relevance. Herein, we describe three model systems with different levels of manipulability and complexity which have been used to uncover molecular mechanisms of interactions. First, we discuss *Bacillus*-*Streptomyces* pairwise interactions to highlight the high experimental control and manipulability of this system used to uncover molecular mechanisms of microbial competition. We then discuss the *Aliivibrio*-squid system, which is uniquely suited for studies of microbial colonization. Finally, we discuss gnotobiotic mice as a model system that can be used to investigate mammalian gut interactions. We highlight where each of these models excels (Fig. 1) and describe limitations within each system to underscore the importance of selecting an appropriate model to address the scientific question at hand.

## UNCOVERING MOLECULAR MECHANISMS OF INTERACTIONS USING *BACILLUS* AND *STREPTOMYCES*

Among the simplest model systems for exploring microbial interactions are pairwise interactions between culturable bacteria. Importantly, these systems intrinsically offer high experimental control to study the molecular underpinnings of interactions that occur between and within microbial communities. As an example, coculture of the soil bacteria Bacillus subtilis and *Streptomyces* spp. demonstrates the power to dissect the molecular mechanisms of competition. Both B. subtilis and *Streptomyces* species are amenable to genetic manipulation, produce antibiotics and other secondary metabolites, and undergo multicellular development (e.g., biofilm formation, motility, and sporulation) on agar plates, providing macroscopic visualization of interactions. Together, the ability to perform mutagenesis screens, generate targeted gene deletions and complements, extract secondary metabolites in isolation, and easily adjust medium and plating configurations to uncover new macroscopic phenotypes all contribute to this system’s high level of experimental manipulability.

**FIG 2:**
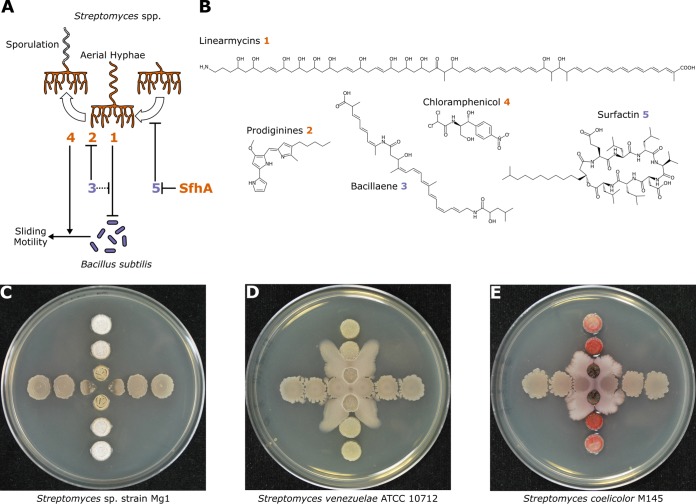
Secondary metabolites mediate interactions between B. subtilis and *Streptomyces* spp. (A) Summary schematic of interactions between B. subtilis and *Streptomyces* spp. The secondary metabolites produced by B. subtilis and *Streptomyces* spp. are represented by the purple and orange numbers, respectively, and the chemical structures are shown in panel B. SfhA refers to surfactin hydrolase produced by *Streptomyces* sp. strain Mg1 that specifically hydrolyzes the ester linkage in surfactin (compound 5). (C to E) *Streptomyces* spp. (vertical) and B. subtilis (horizontal) spotted in a perpendicular pattern on agar plates. (C) B. subtilis colonies proximal to *Streptomyces* sp. strain Mg1 colonies are lysed by linearmycins (compound 1). (Republished from *Frontiers in Microbiology* [3].) (D) Subinhibitory concentrations of chloramphenicol (compound 4) produced by Streptomyces venezuelae induce sliding motility of proximal B. subtilis colonies. (E) Production of the red pigment prodiginine (compound 2) is strongly induced in Streptomyces coelicolor colonies proximal to sliding B. subtilis colonies, which do not produce bacillaene (compound 3). (Images in panels D and E courtesy of Yongjin Liu and Paul Straight, reproduced with permission.)

Pairwise interactions between *Bacillus* and *Streptomyces* demonstrate that secondary metabolites have multiple roles mediating competition (Fig. 2). For instance, B. subtilis produces the lipopeptide surfactin, which triggers its own biofilm formation and multicellular motility (5–7). In contrast, surfactin interferes with the aerial development and sporulation of many *Streptomyces* spp. (8). However, *Streptomyces* sp. strain Mg1 produces a secreted hydrolase that detoxifies surfactin and allows this bacterium to sporulate when cultured with B. subtilis (9). Similarly, B. subtilis produces bacillaene that interferes with prodigiosin pigment production in Streptomyces coelicolor and Streptomyces lividans (10, 11) and protects B. subtilis from lysis by linearmycins produced by strain Mg1 (12–14) (Fig. 2C). In addition to bacillaene, B. subtilis may protect itself from linearmycin-induced lysis by activating a linearmycin-induced, coupled signaling system and exporter that are necessary and sufficient for linearmycin resistance (12, 15). Finally, as an additional means to escape competition, subinhibitory concentrations of chloramphenicol and several other ribosome-targeting antibiotics induce directional sliding motility in B. subtilis away from *Streptomyces* (16) (Fig. 2D).

We highlight the above as examples of multifaceted interactions that can occur between one pair of microbes. Further, even by simply substituting one member of the pair, new interaction dynamics may emerge. For instance, recent work on interactions between Streptomyces venezuelae and Saccharomyces cerevisiae uncovered a new type of “exploration” motility in S. venezuelae induced by the production of volatile trimethylamine (17). However, it is important to consider the artificial abstraction when microbes are transplanted into the laboratory. Compared to microbes in their natural environments, microbes in growth medium encounter atypical nutrients at inordinate concentrations and grow at unnaturally high cell densities. Consequently, microbes may produce extracellular products (e.g., antibiotics) at concentrations that elicit nonphysiological/hormetic responses in interacting partners (18, 19). Furthermore, the evolutionary implications from pairwise interactions are often unknown or unclear. Nevertheless, microbial coculture allows us to infer mechanisms that are impossible to uncover from sequencing studies alone. Therefore, to gain similar mechanistic insight into interactions occurring in communities, model systems where microbes can be isolated in pure culture and investigated in simplified pairwise interactions are invaluable.

## COLONIZATION OF THE LIGHT ORGAN BY ALIIVIBRIO FISCHERI TO INVESTIGATE HOST-MICROBE INTERACTIONS

The bacterium Aliivibrio fischeri (formerly Vibrio fischeri) specifically establishes a symbiosis within the light organ of newly hatched Hawaiian bobtail squid (Euprymna scolopes). This symbiosis has proven an excellent system to investigate colonization dynamics and specificity: though the ocean harbors an incredibly complex microbial community (>10^6^ bacterial cells/ml), the relatively rare A. fischeri (<1 in 5,000 cells) specifically colonizes the light organ (20).

Specialized cilia and mucus recruit *A. fischeri* during early squid development. Bacteria within the mucus are chemotactically attracted toward pores and swim into light organ crypts (21). During the earliest stages of colonization, *A. fischeri* expresses a suite of genes under the “symbiotic colonization-sensor” RscS regulator (22, 23), which promotes polysaccharide production and biofilm formation (24–26) essential for colonization. The bacterially produced, diaminopimelic acid (DAP) type peptidoglycan tracheal cytotoxin (TCT) and lipid A cause apoptosis of ciliated cells (20). The squid subsequently detoxifies TCT (27) and lipid A (28), followed by hemocyte infiltration and tissue regeneration to form the mature light organ (20). Further, squid nitric oxide (NO) signaling (29, 30) and detoxification (31) are tuned in response to colonization, modulating *A. fischeri* populations and excluding competitors from the light organ (20). When RscS is introduced into *A. fischeri* MJ11, a fish symbiont that naturally lacks RscS, the bacteria gain the ability to colonize E. scolopes (23), despite more than 400 unique genes in the laboratory squid strain ES114 compared to MJ11. Aside from biofilm formation and RscS-controlled responses, bacterial motility (20), type VI secretion systems (32), bacterial stress responses (33), other *A. fischeri* regulatory cascades (34), and host genetic factors (35) play key roles in colonization success.

An implicit and unique strength of the squid-*A. fischeri* light organ system is its simplicity, as one-host, one-microbe studies are experimentally manageable and yield ecologically relevant insights into the molecular mechanisms of this symbiosis. Historically, the majority of mechanistic research describing both host and microbe in the squid-*Aliivibrio* symbiosis has focused on a single strain, *A. fischeri* ES114. As such, assessing the extent to which the molecular insights of ES114 colonization apply to other *A. fischeri* strains remains an ongoing effort in this system. Notably, multiple strains of ecologically and phylogenetically distinct *A. fischeri* have been experimentally evolved within the squid host, selecting for alleles of the regulator *binK* that coordinate symbiosis traits and enhance colonization and growth within the light organ (36). Thus, to better understand how specificity relates to the diversity of both *A. fischeri* and *E. scolopes* that exists in nature, future studies are needed to address the impact of strain- and population-level diversity on colonization success and host-microbe fidelity. Nevertheless, the many molecular interactions between one host species and one bacterial strain in this system, even when restricting focus to interactions surrounding colonization, make it a promising research area. Furthermore, whether the specialized physical, chemical, and genetic interactions between squid and *A. fischeri* during colonization have broader implications across different microbes and hosts is unknown. However, a newly emerging system involves the squid nidamental gland, which is situated next to the light organ and harbors a more complex community that consists of *Roseobacter*, *Flavobacteriales*, *Rhizobiales*, and *Verrucomicrobia* (37). We envision that comparison between these two adjacent organs within the same animal that recruit a different set of microbial symbionts from the same seawater environment will provide further insight into how host selection affects microbiome composition and function.

## LEVELS OF COMPLEXITY IN GERMFREE MICE

In humans, the gut microbiota is a complex community containing hundreds of species that impact a variety of health outcomes (38, 39). The microbiota is critical for normal development, as germfree animals possess immune, digestive, and behavioral differences compared to conventional counterparts (40). Germfree animals offer a platform for characterizing interactions with the host and defined communities of microbes (together known as gnotobiotics), ranging from monoassociations to complex communities. Arguably, monocolonized and germfree animals represent vast oversimplification. Defined synthetic communities simplify complex microbiotas while maintaining diversity, and the use of genome-sequenced strains facilitates multi-omics studies (41, 42). Further, using a simplified core microbiota with a genetically tractable strain of interest offers a compromise between creating a well-controlled experiment and not relying on monoassociation studies. For example, to determine the role of the microbial conversion of choline to trimethylamine, mice were colonized with a simplified, six-member gut microbiota containing a single member that could metabolize choline or a mutant of the same strain that was unable to use choline. This approach demonstrates that choline-metabolizing bacteria compete with their hosts for choline and can exacerbate diet-induced metabolic disease in hosts and alter DNA methylation patterns in the brains of offspring (43). Notably, the choline utilization pathway is not taxonomically conserved, and it would be impossible to infer this phenotype from sequencing the 16S rRNA gene from gut communities (44).

To study entire communities, germfree mice can be colonized with complex communities, often from fecal samples. Donor communities can demonstrate a proof of principle of microbiota-mediated effects on a particular phenotype, such as linking the microbiota to obesity (45, 46). However, with increasing community complexity, more reproducibility issues arise. For instance, though donor communities reduce the artificial nature of gnotobiotics, rare strains may be stochastically lost in the transplanted community. When human fecal microbiota are transplanted into germfree mice, 10 to 30% of operational taxonomic units fail to colonize the mouse (47). Strains present at 0.15% of the community can impact phenotypes like choline conversion to trimethylamine (44). Alternatively, using donor microbiota derived from the same species as the germfree animal can be more appropriate for certain ecological questions and better retain members (48). Reproducibility is also an issue for studying some emergent phenotypes of complex communities, as maintenance of certain members may depend on diet or even water pH (49), and social, coprophagous animals like mice may necessitate cages as biological units of replication, rather than individuals (50, 51). Though reproducibility issues also arise in simplified communities, troubleshooting whether small changes in abiotic or biotic factors influence phenotypes is more challenging in complex communities and could be limiting in a mouse system with a relatively slow generation time and ethical constraints on animal usage.

Overall, gnotobiotic animals provide an approach to interrogate the role of complex microbiota in emergent phenotypes of interest by reducing the complexity to controllable independent variables (e.g., a single bacterial strain or product). Experimenting with multiple levels of community complexity applies to germfree hosts beyond mice (e.g., *Arabidopsis*, *Danio*, and *Drosophila*), but specific mechanisms may differ. For example, facultatively anaerobic pathogens exploiting inflammation-associated oxidation in the typically anaerobic mouse gut would not be readily apparent in aerobic *Drosophila* guts (52, 53). Further, although mice are often sought as medically relevant models, the ease of producing large numbers of gnotobiotic animals and availability of tools in other models, such as imaging in translucent zebrafish, can reveal alternative mechanisms for microbial proteins mediating mutualism that may have remained obscure in a mouse model (54). Ultimately, shared insights from different models support broad ecological principles of microbiome interactions.

## CONCLUSION

By leveraging the unique features of experimental microbiome systems, important and outstanding questions can be addressed (Fig. 1). Chief among these questions is understanding how interactions between microbes and hosts influence behavior and health and how communities respond to perturbations, such as invasion or abiotic stresses. Although it is well understood that microbiomes influence the health of hosts and macroscopic ecosystems, the specific molecular mechanisms remain elusive. For instance, what interactions differentiate “healthy” and “dysbiotic” microbial communities are often unresolved. Further, communities can exhibit emergent phenotypes that are not seen when members are grown in isolation, such as catabolism of recalcitrant materials (55, 56), biofilm formation (57), or antibiotic production (58–60).

As microbiome research continues, new frameworks for characterizing the interactions that occur within microbial communities will emerge from novel systems spanning the spectra of complexity and tractability and developments enabling established systems to address new questions. As examples, two particular systems that we are especially interested in are the cheese rind microbial community and the gardens of fungus-growing ants. The cheese rind microbial community is an emerging system particularly suitable for characterization of multipartite interactions and simulating ecological phenomena through control of abiotic factors (61–64), yet the unclear evolutionary relationships between members may limit its applicability to coevolved, natural systems. In contrast, because the microbial symbionts of fungus-growing ants provide a coevolutionary framework from which to investigate microbial population dynamics (65, 66), nutrient flow (67), host-pathogen interactions (68–70), and defensive symbiosis (71), further characterizations of these microbiomes may provide broader implications for other natural systems (59, 60).

In conclusion, delineating community states that contribute to emergent properties and complex interactions will require experimental models, and the ideal balance between a model’s complexity, ease of manipulation, and overall biological relevance will depend upon the scientific questions posed.
